# Editorial for the Special Issue on Advances in Microfluidics for Quantifying Cell Mechanics and Biotransport

**DOI:** 10.3390/mi13071127

**Published:** 2022-07-17

**Authors:** Hiroaki Ito, Naoki Takeishi

**Affiliations:** 1 Department of Physics, Graduate School of Science, Chiba University, Yayoi-cho 1-33, Inage-ku, Chiba 263-8522, Japan; 2 Graduate School of Engineering Science, Osaka University, 1-3 Machikaneyama, Toyonaka 560-8531, Japan

Microfluidics is a powerful tool to precisely control fluids as well as to manipulate suspended small particles in a micrometer-sized space. Numerous efforts have been made to develop this technology in interdisciplinary fields including physics, engineering, medicine, biology and chemistry. The efforts in these fields have enabled us to perform automated, high-throughput, and precisely controlled assays to treat micro-objects such as microdroplets, microparticles, and single cells, even with extremely small amounts of samples. Owing to the advantages of microfluidics, clarifying the mechanisms of the microscopic biological phenomena has been one of the central topics in these fields. In this Special Issue, we focused on recent microfluidic approaches that shed light on the biological mechanics and transport phenomena, especially on the deformation and transportation of passive micro-objects exposed to shear stress and behavioral dynamics of bacteria as active microswimmers. This Special Issue contains seven research papers covering different subjects related to cell mechanics and biotransport. Three of the research papers report the experimental approaches, and four of the papers focus on numerical investigations.

Lee et al. [1] reported on novel droplet-based microfluidics to form microparticles with front–back asymmetry in their shapes. With the proposed step, aqueous microdroplets containing sodium alginate deform into a front–back asymmetric shape through fusion with another droplet containing calcium ions, where the location of the fusion was controlled around the stagnation point on the droplet surface. The findings lead not only to potential applications to various micromachines and drug carriers but also to potential understanding of the behaviors of deformable micro-objects and polar microswimmers under microfluidic shear flow.

Okuyama et al. [2] experimentally investigated the spatial population dynamics of ciliate by combining the image processing of 3D micrographs and machine-learning-assisted quantification. They measured the 3D spatial distribution of ciliate and its temporal evolution, and revealed that cells tend to accumulate on both the solid–wall surface and air–water interface, which are potentially preferable conditions to survive, i.e., conditions rich in feed and oxygen, respectively, in a biological aspect. The characteristic cell distribution is theoretically explained by Fick’s law with appropriate boundary conditions. This result bridges the gap between the single-cell dynamics of ciliate on a microscopic scale and its collective behaviors on a mesoscopic scale.

Sugihara-Seki and Takinouchi [3] conducted in vitro experiments to investigate the cross-sectional distribution of platelet-sized particles in human red blood cell (RBC) suspension flowing through square microchannels. Fluorescence microscope observations showed that the particles are concentrated near the corners of the cross section rather than uniform margination along the entire circumference of the cross section. The concentrating of particles near the corners was more enhanced for higher hematocrits. These results will help us to design microfluidic devices, which apply the segregation phenomenon to the separation or sorting of suspended particles or cells in multicomponent suspensions flowing through rectangular channels.

Takeishi et al. [4] numerically investigated the dynamics of RBCs flowing in a microtube with diameters of 15 μm, with different capillary numbers, which is the ratio between the fluid viscous force and membrane elastic force. The authors found that RBCs did not always exhibit axial migration even though the flow was assumed to be almost inertia-less. Furthermore, their numerical results demonstrated that the axial or nonaxial migration of RBCs depended on the stable deformed RBC shapes, and the equilibrium radial position of the RBC centroid correlated well with energy expenditure associated with membrane deformation.

Yamashita et al. [5] numerically investigated the inertial focusing of a rigid spherical particle flowing in a rectangular duct with an aspect ratio of the cross-section *A* = b/h = 2 (*b*: long side width; *h*: short side width), and elucidated the variation of the inertial focusing depending on the Reynolds number of 50 to 400. Simulations covered blockage ratios d/h from 0.1 to 0.3 (*d*: a particle diameter). The authors accurately estimated the particle trajectories based on the lift map and Stokes drag, and identified the particle-focusing positions that appeared in the cross-section.

Otomo and Kira [6] theoretically and numerically investigated the behavior of microparticles when passing through a fibrous bed, which is a layered internal structure. The flow was assumed to be a Stokes flow, and the Stokesian dynamics method was used. Simulations considered five different types of structures characterized by a monolayer with fiber volume fractions. The simulations showed that the behavior of individual micropaticles varied depending on the internal structures, while the average permeation velocity was primarily determined by the fiber volume fraction.

Moreau and Ishimoto [7] theoretically and numerically assessed the controllability and optimal control properties of a spherical swimmer controlled by the modulation of an oscillatory flow generated by a wall and described the key features of the optimal control for motion along the wall, notably its periodicity. The results on how oscillatory flows can be used to boost a microswimmer’s speed could also help to understand in more depth the mechanisms in biological contexts featuring microorganisms swimming near active walls.

In summary, the seven original papers in this Special Issue highlight the recent advances on the deformation, transportation, and behavioral dynamics of passive and active micro-objects, towards the clarification of biological mechanics and transport. We, the Guest Editors, believe that further understanding and the clarification of mechanisms of the microscopic biological phenomena will be provided through the cooperation of experimental, theoretical, and numerical efforts such as those included in this Special Issue. We hope that the Special Issue will be a good starting point for future studies in this field. Finally, the Guest Editors would like to acknowledge all the researchers who contributed their original papers, reviewers who provided fruitful comments, and the members of the editorial office for their valuable time and efforts.
